# Ecological surveillance of bat coronaviruses in Sarawak, Malaysian Borneo

**DOI:** 10.1186/s13104-021-05880-6

**Published:** 2021-12-20

**Authors:** Cheng-Siang Tan, Vaenessa Noni, Jaya Seelan Sathiya Seelan, Azroie Denel, Faisal Ali Anwarali Khan

**Affiliations:** 1 Faculty of Medicine and Health Sciences, Universiti Malaysia Sarawak, Kota Samarahan, Sarawak Malaysia; 2 Institute for Tropical Biology and Conservation, Universiti Malaysia Sabah, Kota Kinabalu, Sabah Malaysia; 3 Sarawak Forestry Corporation, Kota Sentosa, Sarawak Malaysia; 4 Faculty of Resource Science and Technology, Universiti Malaysia Sarawak, Kota Samarahan, Sarawak Malaysia

**Keywords:** Bat, Cave, Coronavirus, Guano, Sarawak

## Abstract

**Objective:**

Coronaviruses (CoVs) are natural commensals of bats. Two subgenera, namely Sarbecoviruses and Merbecoviruses have a high zoonotic potential and have been associated with three separate spillover events in the past 2 decades, making surveillance of bat-CoVs crucial for the prevention of the next epidemic. The study was aimed to elucidate the presence of coronavirus in fresh bat guano sampled from Wind Cave Nature Reserve (WCNR) in Sarawak, Malaysian Borneo. Samples collected were placed into viral transport medium, transported on ice within the collection day, and preserved at − 80 °C. Nucleic acid was extracted using the column method and screened using consensus PCR primers targeting the RNA-dependent RNA polymerase (RdRp) gene. Amplicons were sequenced bidirectionally using the Sanger method. Phylogenetic tree with maximum-likelihood bootstrap and Bayesian posterior probability were constructed.

**Results:**

CoV-RNA was detected in ten specimens (47.6%, n  = 21). Six alphacoronavirus and four betacoronaviruses were identified. The bat-CoVs can be phylogenetically grouped into four novel clades which are closely related to Decacovirus-1 and Decacovirus-2, Sarbecovirus, and an unclassified CoV. CoVs lineages unique to the Island of Borneo were discovered in Sarawak, Malaysia, with one of them closely related to Sarbecovirus. All of them are distant from currently known human coronaviruses.

## Introduction

Coronaviridae consists of a large family of viruses that cause infections and diseases in a large range of vertebrates. Coronaviruses (CoVs) can be classified into four genera, *Alphacoronavirus*, *Betacoronavirus*, *Gammacoronavirus*, and *Deltacoronavirus*. Both *Alphacoronavirus* and *Betacoronavirus* houses CoVs of public health importance. Currently, there are seven known human CoV types, 2002/03 Severe Acute Respiratory Syndrome Coronavirus (SARS-CoV), 2012 Middle Eastern Respiratory Syndrome CoV (MERS-CoV), 2019 SARS-CoV-2, and four seasonal CoVs (HCoV-229E, HCoV-NL63, HCoV-OC43 and HCoV-HKU1) in which nearly all of them can be traced to their bat origin [1, 2]. The discovery of two bats CoVs, KSA-287 from *Taphozous perforatus* and RaTG13 from *Rhinolophus affinis* with 100% and 96% sequence identity to MERS-CoV and SARS-CoV-2 respectively, sealed the hypothesis that these CoVs of Public Health Emergency of International Importance (PHEIC) indeed have originated from bats. However, the progenitor for SARS-CoV was never discovered.

The SARS-CoV epidemic started in Guangdong, China in 2002, rampaging through numerous countries for nine months, infecting 8096, claiming the lives of 774. A decade later, MERS-CoV emerged in the Kingdom of Saudi Arabia, but cases have mostly been reported around the Middle East, Africa and South Asia with occasional exported cases [3]. As of June 2021, there were 2564 confirmed cases of MERS, including 886 associated death, with the case-fatality-rate (CFR) of 34.4%[4]. Later, a novel beta-coronavirus named SARS-CoV-2 was discovered in a cluster of patients with acute viral pneumonia of unknown origin in Wuhan, China. However, SARS-CoV-2 has a relatively low CFR of 2.1% compared to SARS-CoV and MERS-CoV but due to its high transmissibility, it has resulted in more than 208 million infections and 4.4 million deaths worldwide (as of 17th August 2021) [5].

Bats from the order Chiroptera, including microbat families Rhinolophidae, Hipposideridae, Emballonuridae, Miniopteridae, and Vespertilionidae are known to be the reservoir of bat CoVs in Asia [2]. These competent hosts can also be found in the touristic Wind Cave Nature Reserve (WCNR) in Bau District, Sarawak, Malaysian Borneo [6].

Other virological surveillance in Sarawak, Malaysia mainly revolves around viruses of public health importance, but the study on bat viruses in Gunung Mulu National Park, Sarawak (4°02′60.00′′ N 114°55′58.80′′ E), has led to the identification of *Sarawak* *mobatvirus*, a novel hantavirus within the genus *mobatvirus* [7]. Therefore, in the wake of the COVID-19 pandemic, it is timely that virological surveillance should be conducted to determine the presence and diversity of bat-CoVs in bat populations in Sarawak.

## Main text

### Methods

**Fig. 1:**
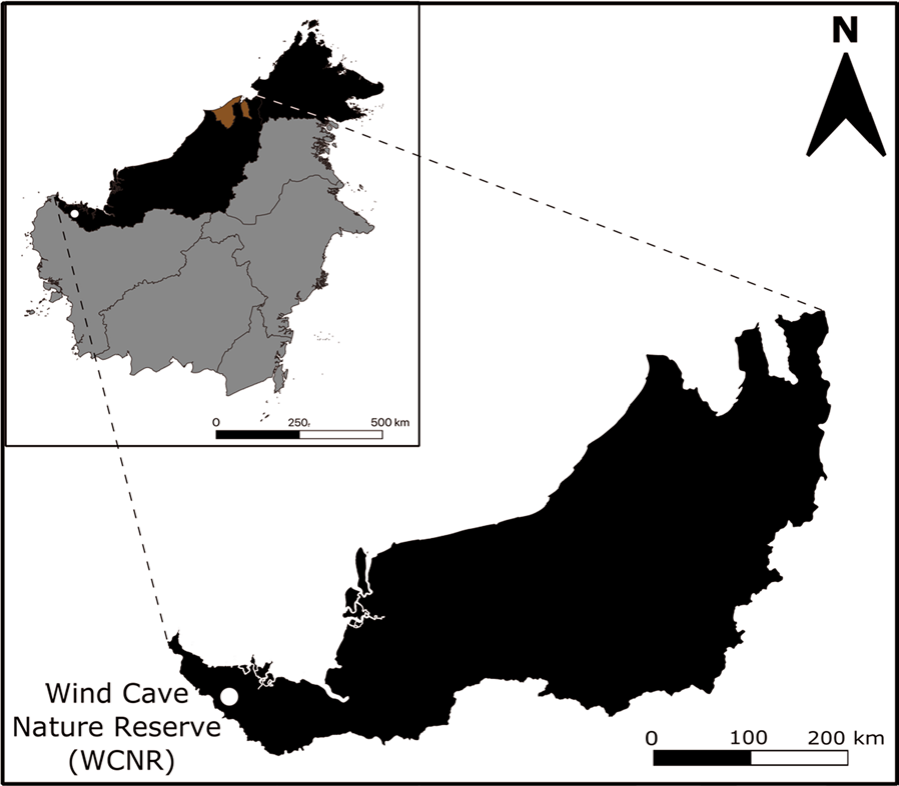
Sampling location at Wind Cave Nature Reserve (WCNR), Sarawak, Malaysia Borneo. Three countries found on the Island of Borneo are differentiated with different colors: Black, East Malaysia; brown, Brunei; and grey, Kalimantan, Indonesia. The White dot represents the location of WCNR (1°24′55′′ N 110°08′7′′ E) where guano specimens were sampled for virological surveillance. Map was created in QGIS Version 3.20.3 (http://qgis.org/en/site/)

WCNR is located 1º24′55′′ N 110º08′7′′ E (Fig. 1). We have collected 21 pooled bat guano from WCNR on March 8th, 2021 and screened them for the presence of the partial RNA-dependent RNA polymerase gene of coronavirus. Five (5) pellets of fresh guano from insectivorous bats collected on a plastic tarp were pooled in 500 μL of ice-cold viral transport medium (1st Base), vortex-mixed and centrifuged at 13,000×*g* at 4 °C. Supernatant was used for nucleic acid extraction using High Pure Viral Nucleic Acid Extraction Kit (Roche). For phylogenetic analysis, the 440-bp gene of coronavirus was amplified using family-wide hemi-nested RT-PCR using the modified Watanabe primers: CoV-RVS3 5′-CCATCATCASWYRAATCATCATA-3′, CoV-FWD3 5′-GGTTGGGAYTAYCCHAARTGTGA-3′ and CoV-FWD4/Bat 5′-GAYTAYCCHAARTGTGAYAGAGC-3′. The complementary DNA synthesis was performed at 42 °C for 60 min using RevertAid Reverse Transcriptase (ThermoScientific) using CoV-RVS3. The conditions for the first round PCR were 2 min at 94 °C; 40 cycles for 20 s at 94 °C, 30 s at 50 °C, 30 s at 72 °C; 1 min. The conditions for the nested-PCR are identical except for the higher annealing temperature at 59 °C. PCR amplicons were resolved in 2% agarose buffered in 1X TBE (1st Base) supplemented with 10 μg/mL ethidium bromide (Promega), excised, and sequenced using the BigDye terminator v3.0. Amplicons were sequenced bidirectional using CoV-FWD3 and CoV-FWD4/Bat, amplicons with mixed or poor sequence read were ligated into pJet2.1 vector and transformed into chemically competent *Escherichia coli* DH5α (ThermoScientific) and sequenced using the vector primers (1st Base) [8]. Amplicon sequences were analysed with the Basic Local Alignment Search Tool software (BLAST) at the National Centre for Biotechnology Information (NCBI) to determine their corresponding species followed by nucleotide identity analysis using Sequence Identity and Similarity (SIAS) (http://imed.med.ucm.es/Tools/sias.html).

Sequence alignment of amplicon sequences and available CoV RdRp sequences from GenBank was carried out using MAFFT (https://www.ebi.ac.uk/Tools/msa/mafft/). Phylogenetic tree was constructed based on 348 bp RdRp gene sequence with maximum-likelihood bootstrap/Bayesian posterior probability (ML_b_/PP_v_) support using 1000 bootstraps and 1,00,00,000 mcmc generations respectively. The virus subgenus in the ML tree are categorized based on ICTV-approved genomes of all known Alpha (HKU10 isolate (MN312307), Alpha/R.fer/HuBCH/2013 (KJ473807), PEDV CV777 (AF353511), BtCoV/NL63-9b (KY073745), HCoV-NL63 (NC0052645), HCoV-299E (NC002645), MkCoV/WD1127 (HM245925), CCoV/Human/SwkMY/2018 (MW591993), Alpha/Miniopterus/TH/B311-3/2008 (KJ020586) and BtCoV-HKU8/AFCF77(EU420139) and Beta-coronaviruses (Beta/Rousettus/CH/GCCDC1-358/2014 (KU762338), MurineCoV-A59 (AY700211), HCoV-OC43 (AY391777), BetaCoV-HKU24-R05005I (KM349742), BtCoV-HKU5 (EF065509), HCoV-EMC (JX869059), BtCoV-HKU4 (EF065505), SARS-CoV-2 Wuhan-Hu-1 (MN908947), SARS-CoVr-2 RaTG13 (MN996532), SARS-CoV-1 Tor2 (AY274119), BtCoV-SARSlike-CoV/SL-CoVXC45 (MG772933) and Beta/H.pra/ZhejiangCH/2013 (KF636752).

### Results

**Fig. 2:**
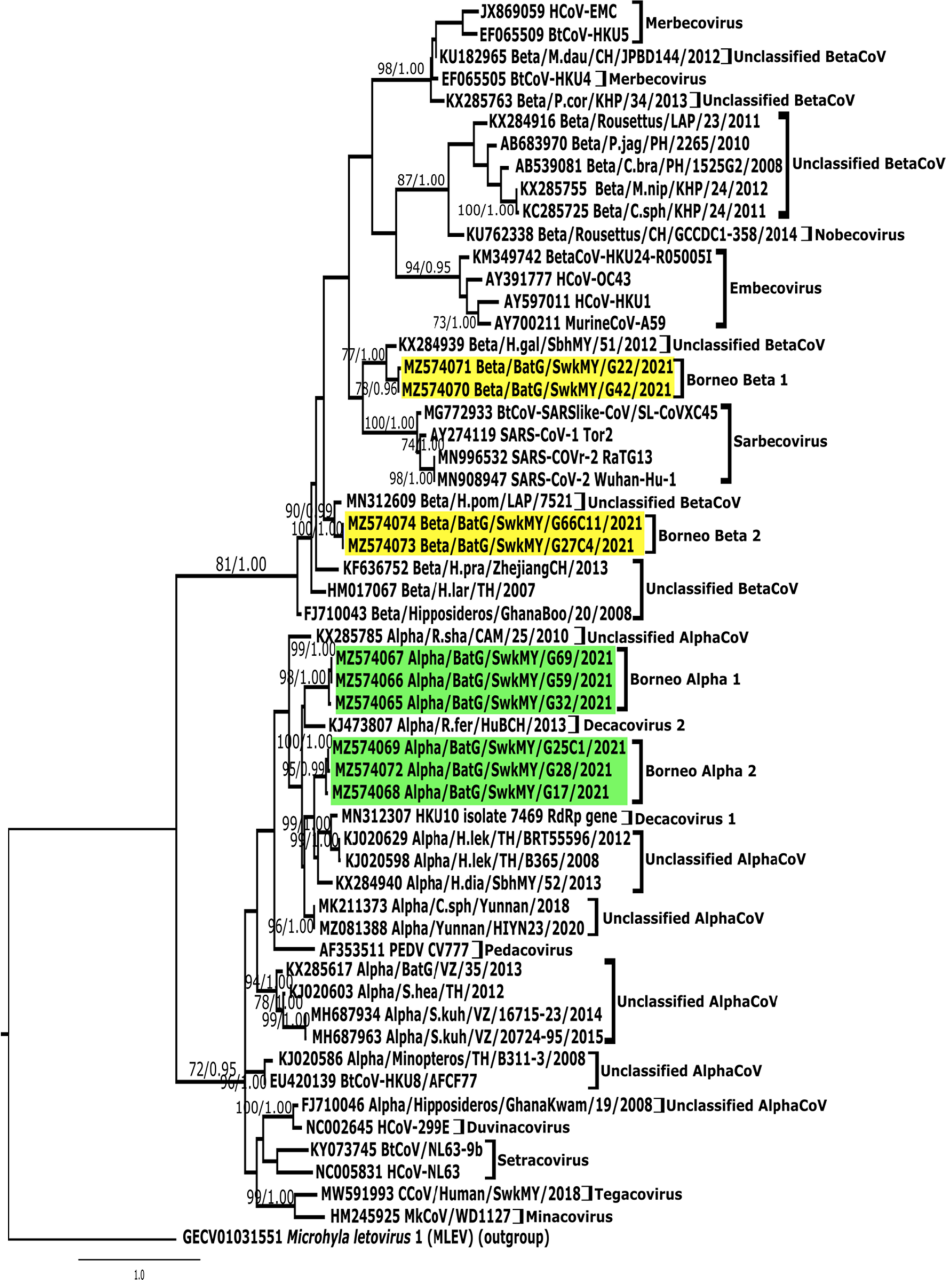
Phylogenetic tree of novel coronaviruses based on the nucleotide sequence of the partial RdRp gene. Maximum-likelihood tree of 348 bp fragment of the RdRp gene from bat CoVs found in this study are colored according to their subgenera (Yellow  = Borneo Beta-1 and Borneo Beta-2; Green  = Borneo Alpha-1 and Borneo Alpha-2). *Microhyla letovirus* 1 (MLeV) (GVEC01031551) was used as the outgroup. Individual nodes are considered well supported when ML bootstrap values (BS) are at least 70% and Bayesian inference (PP) are at least 0.95% and tree with the highest log likelihood is shown (− 8794.58). Taxa are named according to the following pattern: Accension Number CoV Genus/Source or host/Country/Lab code/Year. The following are the names in full of species, M.nip, *Megaerops niphanae*; C.sph, *Cynopterus sphinx*; C.bra, *Cynopterus brachyotis*; P.jag, *Ptenochirus jagori*; M.dau, *Myotis daubentonii*; P.cor. *Pipistrellus coromandra*; H.pom, *Hipposideros pomona*; H.gal, *Hipposideros galeritus*; H.pra, *Hipposideros pratti*; H.lar, *Hipposideros larvatus*; H.dia, *Hipposideros diadema*; H.lek, *Hipposideros lekaguli*; R.hip, *Rhinolophus hipposideros*; R.fer, *Rhinolophus ferrumequinum*; R.sha, *Rhinolophus shameli*; S.kuh, *Scotophilus heathii* and S.hea, *Scotophilus heathii*. Accension Numbers of positive bat guano samples are MZ574065–MZ574074

The PCR positive rate was 47.6% (10/21) and with the distribution of 60% (n  = 6/10) and 40%; (n  = 4/10) alpha and beta-coronaviruses respectively. Bat CoVs identified in this study formed four novel monophyletic clades referred to as Borneo Beta-1 (GenBank: MZ574070, MZ574071), and Borneo Beta-2 (MZ574073, MZ574074), and Borneo Alpha-1 (MZ574065-MZ574067) and Borneo Alpha-2 (MZ574068, MZ574069, MZ573072) (Fig. 2).

Blast analysis revealed that Borneo Alpha-1 and Borneo Alpha-2 are most related to Alpha/Yunnan/HlYN23/2020 (MZ081388) (81.96–83.76% nt identity) and Alpha/H.dia/SbhMY/52/2013 (KX284940) (84.43–85.01% nt identity) from China and Sabah (North Borneo) respectively. Whereas, Borneo Beta-1 and Borneo Beta-2 are most closely related to Beta/H.gal/SbhMY/51/2012 (KX284939) (86.5–87.5% nt identity) and Beta/H.pom/LAP/7521 (MN312609) (89.7–90.4% nt identity) from Sabah state and and Laos respectively [2, 9]. Phylogenetic analysis revealed the close relationship of Borneo Alpha-1 and Borneo Alpha-2 with Decacovirus, specifically Decacovirus-1 and Decacovirus-2 respectively with strong ML_b_/PP_v_ support. On the other hand, Borneo Beta-1 is monophyly to Sarbecovirus, while Borneo Beta-2 is related to a currently uncategorised betacoronavirus.

### Discussion

Despite the small sample size, the findings from this study confirm the presence of CoVs in nearly half of the bat guano sampled in WCNR, Sarawak, Malaysia. This supports the argument that bat guano is a good surrogate for Bat CoV surveillance without the need for life animal capture [10].

All of these CoVs were highly related to CoVs that were previously found in *Hipposideros* spp. suggesting that *Hipposideros* could potentially be their preferred natural host, but these viruses were also found in other bat species such as *Rhinolophus*, *Miniopterus*, *Taphozous*, *Myotis*, *Scotophilus*, and *Cynopterus* (fruit bats) [11–14]. However, SARS-CoV-2 related viruses were mainly identified in *Rhinolophus* bats such as *R. affinis* and *R. malayanus* in Yunnan, China, *R. shameli* in Northern Cambodia and *R. acuminatus* in Eastern Thailand [15].

Of the four novel clades, Borneo Beta-1 is probably of a greater concern as two members from its sister clade are responsible for two pandemics within the past two decades. Nonetheless, Borneo Beta-1 viruses are found to be distant to the SARS-CoVr-2 RaTG13 (MN996532) (69.1–69.7% nt identity), the proposed progenitor of the pandemic SARS-CoV-2 identified in *Rhinolophus affinis* [16]. Further investigations to determine its receptor use should be carried out to assess the risk of spillover from bats to humans [17].

Caves are haven for ecotourism but may also serve as hotspots for bat CoV zoonosis since other sympatric wildlife is present and physical contact with virus-contaminated guano is nearly unavoidable. Although it is widely accepted that an intermediary host is almost always required for efficient spillover of Bat CoV to humans [18, 19], the discovery of CoV strain RaTG13 in Mojiang, Yunnan, China where fatal SARS-like epidemic in 2012 did not discount the possibility of direct spillover [20]. Following three CoVs zoonoses in the past 2 decades, ecotourism and research pertaining to caves and bats should be revisited.

There have been no records of bat CoV zoonoses in Sarawak. Nevertheless, it is important to note that clinical diagnostics of potential SARS-like/Influenza-like respiratory diseases in Sarawak had historically involved only bacterial diagnosis [21]. Virological surveillances were rarely carried out except through external research collaboration that would not impact patient care. Despite the record adoption of real-time molecular assays in the healthcare settings during the SARS-CoV-2 pandemic, it was also shown that real-time assay was inferior to pan-CoV assay in the detection of seasonal HCoV, missing out on many positives, including the novel canine-CoVs in the human respiratory specimens [22, 23]. This is not surprising as real-time primers and probes tend to be more specific as compared to the degenerate primers used in most pan-CoV assays that increased the probability of detecting all circulating CoV genera. Do bear in mind that primer designs are largely based on the skewed representation of viral sequences obtained from other geographical locations, disregarding on the dispersal and vicariance phenomenon. To this, all SARS-like illnesses should be screened using the pan-CoV assay in hope of identifying the potential spillover rapidly and stop the epidemic at the source in a timely manner.

### Conclusions

The bats in WCNR harbour rich diversity of both alpha-coronaviruses and beta-coronaviruses that are unique to Borneo Island. Although these novel CoVs are not closely related to any seasonal, epidemic, and pandemic CoVs; visitors that are at risk of being in direct contact with virus contaminated guano. Basic biosafety precautions should be practised to minimize the possibility of spillover.

## Limitations

The study was based on a small sample size obtained in a restrictive condition during the SARS-CoV-2 pandemic. Increasing the sample size may potentially lead to the discovery of more novel CoVs or quasispecies of the CoVs reported in this study. The partial RdRp sequence analysis may have suggested the novelty of the CoVs discovered in this study but longer sequence information on other genes and hopefully whole genomic sequences may enhance the current findings or suggest otherwise.

## Data Availability

All bat CoV nucleotide sequences obtained in this study can be accessed in GenBank (https://www.ncbi.nlm.nih.gov/) under the Accession Number MZ574065-MZ574074.

## Abbreviations

CoV: Coronavirus
MERS: Middle eastern respiratory syndrome
nt: Nucleotide
PCR: Polymerase chain reaction
PHEIC: Public health emergency of international importance
RNA: Ribonucleic acid
SARS: Severe acute respiratory syndrome
WCNR: Wind cave nature reserve
